# T14diLys/DOPE Liposomes: An Innovative Option for siRNA-Based Gene Knockdown?

**DOI:** 10.3390/pharmaceutics17010025

**Published:** 2024-12-27

**Authors:** Sophie Meinhard, Frank Erdmann, Henrike Lucas, Maria Krabbes, Stephanie Krüger, Christian Wölk, Karsten Mäder

**Affiliations:** 1 Department of Pharmaceutical Technology, Faculty of Natural Sciences I, Institute of Pharmacy, Martin Luther University Halle-Wittenberg, Kurt-Mothes-Str. 3, 06120 Halle/Saale, Germany; sophie.meinhard@pharmazie.uni-halle.de (S.M.); henrike.lucas@pharmazie.uni-halle.de (H.L.); 2 Department of Pharmaceutical Pharmacology and Toxicology, Faculty of Natural Sciences I, Institute of Pharmacy, Martin Luther University Halle-Wittenberg, Kurt-Mothes-Str. 3, 06120 Halle/Saale, Germany; frank.erdmann@pharmazie.uni-halle.de; 3 Research Center for Drug Therapy Halle, Martin Luther University Halle-Wittenberg, Wolfgang-Langenbeck-Str. 4, 06120 Halle/Saale, Germany; 4 Pharmaceutical Technology, Medical Faculty, University of Leipzig, Eilenburger Strasse 15A, 04317 Leipzig, Germany; maria.krabbes@medizin.uni-leipzig.de (M.K.); christian.woelk@medizin.uni-leipzig.de (C.W.); 5 Biocenter, Microscopy Unit, Martin Luther University Halle-Wittenberg, Weinbergweg 22, 06120 Halle/Saale, Germany; stephanie.krueger@biozentrum.uni-halle.de

**Keywords:** siRNA, lipoplex, ionizable lipid, T14diLys, extrusion, transfection

## Abstract

Background/Objectives: Bringing small interfering RNA (siRNA) into the cell cytosol to achieve specific gene silencing is an attractive but also very challenging option for improved therapies. The first step for successful siRNA delivery is the complexation with a permanent cationic or ionizable compound. This protects the negatively charged siRNA and enables transfection through the cell membrane. The current study explores the performance of the innovative, ionizable lipid 2-Tetradecylhexadecanoic acid-(2-bis{[2-(2,6-diamino-1-oxohexyl)amino]ethyl}aminoethyl)-amide (T14diLys), in combination with 1,2-dioleoyl-*sn*-glycero-3-phosphoethanolamine (DOPE), for siRNA delivery and the impact of the production method (sonication vs. extrusion) on the particle properties. Methods: Liposomes were produced either with sonication or extrusion and characterized. The extruded liposomes were combined with siRNA at different N/P ratios and investigated in terms of size zeta potential, encapsulation efficiency, lipoplex stability against RNase A, and knockdown efficiency using enhanced green fluorescent protein (eGFP)-marked colon adenocarcinoma cells. Results: The liposomes prepared by extrusion were smaller and had a narrower size distribution than the sonicated ones. The combination of siRNA and liposomes at a nitrogen-to-phosphate (N/P) ratio of 5 had optimal particle properties, high encapsulation efficiency, and lipoplex stability. Gene knockdown tests confirmed this assumption. Conclusions: Liposomes produced with extrusion were more reproducible and provided enhanced particle properties. The physicochemical characterization and in vitro experiments showed that an N/P ratio of 5 was the most promising ratio for siRNA delivery.

## 1. Introduction

Since the discovery of RNA Interference (RNAI) in 1998 [1], the development of small interfering RNA (siRNA) delivery systems has become an exciting field for the treatment of previously untreatable diseases. In particular, the first six siRNA therapeutics ONPATTRO^®^ (patisiran) [2], GIVLAARI™ (givosiran) [3], OXLUMO^®^ (lumasiran) [4], LEQVIO^®^ (inclisiran) [5], RIVFLOZA^®^ (nedosiran) [6], and AMVUTTRA^®^ (vutrisiran) [7], which were approved by the Food and Drug Administration (FDA), show the possible clinical application for siRNA therapy [8]. Besides micro RNA, siRNA is the tool for RNAI, which can silence certain gene expressions by cleaving the target messenger RNA [9]. However, there are several challenges in the formulation process to obtain active siRNA at the target. Naked siRNA cannot be easily applied because it is rapidly degraded by endogenous nucleases, and the negative charge and high molecular weight make it difficult to cross the cell membrane [10,11]. Additionally, rapid elimination by immune system recognition and fast renal clearance are a problem [8,12]. A commonly used method to overcome these challenges is the complexation of siRNA with non-viral carriers, e.g., cationic polymers, polypeptides, or cationic lipids, which could be permanently cationic or ionizable [9,13,14]. Non-viral carriers are preferred to viral ones due to lower immunogenic reactions [15,16,17]. The goal is the development of formulations with high efficacy and low toxicity, which can be used in vitro and in vivo. Today, ionizable lipids are applied since they are less toxic than the permanent cationic lipids used in the past [9]. In this study, ionizable liposomes as non-viral carriers were used and composed of a new ionizable lipid 2-Tetradecylhexadecanoic acid-(2-bis{[2-(2,6-diamino-1-oxohexyl)amino]ethyl}aminoethyl)-amide (T14diLys) and a commonly used helper lipid 1,2-dioleoyl-*sn*-glycero-3-phosphoethanolamine (DOPE) in a molar ratio of 1:2 [18,19,20,21,22]. In previous studies, this ratio was found to have the highest DNA transfection efficiency in human lung carcinoma cells (A549), African green monkey kidney cells (COS-7), and human hepatocellular carcinoma cells (Hep-G2) [19]. When positively charged liposomes are combined with siRNA, they can form lipoplexes [23]. These can protect the siRNA from degradation and enable transfection and gene knockdown in the cytosol due to endocytic internalization [24]. The ionizable lipid T14diLys has been recently developed by Wölk and coworkers and was a hit of an internal screening [18]. It has four primary amine groups that can be protonated to achieve a positive charge. This lipid was combined with DOPE to increase the cellular uptake of DNA-loaded lipoplexes [18]. The T14diLys/DOPE formulations with DNA showed excellent biocompatibility tested with the Hen’s egg test on the Chorioallantonic Membrane test (HET-CAM), and no charge-related toxic effects of the lipoplexes at the nitrogen-to-phosphate ratio (N/P ratio) 4 were observed in vitro. When serum was added to the lipoplexes, the DNA was efficiently protected from degradation, and the particle size remained stable [18,19]. The current study has the following goals: First, we aim to investigate whether these effects could also be transferred to siRNA application with this innovative lipid composition, because it is not possible to transfer the results of DNA studies one-to-one to siRNA. This can be explained by variations of physicochemical properties, such as size, complex formation, and the stability of complexes [25,26,27]. That is why a rescreening of the formulations for siRNA is needed. The liposomes were previously produced by sonication and had a usable, but not optimal size distribution. As a second point, we therefore examined extrusion as an alternative method for particle production. The liposomes and lipoplexes obtained were physicochemically characterized by determining the size, zeta potential, and N/P ratio. In addition, the encapsulation efficiency was examined with the RiboGreen-Assay and the lipoplex stability against RNase A with agarose gel electrophoresis. Whether the sterile filtration of the liposomes with different filter materials affected the transfection efficiency was also investigated. Finally, the N/P ratio with the highest transfection efficiency was determined using an enhanced green fluorescent protein (eGFP) expressing a cell clone from the human colon adenocarcinoma cell line (DLD1), and the cytotoxicity was tested on mouse and human fibroblasts.

## 2. Materials and Methods

### 2.1. Materials

DOPE was obtained from Lipoid (Ludwigshafen, Germany). T14diLys was synthesized in the group of Wölk from the pharmaceutical technology (Medical Faculty, Leipzig, Germany), as previously described [18]. Allstars negative control siRNA (Cat# 1027281) was bought from Qiagen (Venlo, The Netherlands) and Silencer^TM^ GFP (eGFP) siRNA (Cat# AM4626), Lipofectamine2000^TM^ transfection reagent, OPTI-MEM^®^ Reduced Serum Medium, RNase A (10 mg/mL) (DNase and protease free), RiboLock RNase Inhibitor (40 U/µL), and GeneRuler Ultra Low Range DNA Ladder from Thermo Fisher Scientific Inc. (Waltham, MA, USA). Agarose Standard, Glycerin, Chloroform, Polyvinylidene fluoride (PVDF) filter 0,22 µm, Propidium iodide (PI), and 2-(4-Morpholinyl)ethanesulfonic acid (MES) buffer were purchased from Carl Roth GmbH & Co. KG (Karlsruhe, Germany). Minisart^®^ Regenerated Cellulose (RC) filters 0,2 µm were bought from Sartorius Stedim Biotech GmbH (Göttingen, Germany). Heparin sodium, Resazurin, Rosewell Park Memorial Institute Medium 1640 (RPMI Medium), Dulbecco’s modified Eagle Medium (DMEM), Penicillin/Streptomycin (Pen/Strep), Fetal Bovine Serum (FBS), Dulbecco’s phosphate-buffered saline, Trypsin-EDTA, Triton^TM^ X100, and 20× Tris-acetate-EDTA (TAE) buffer were obtained from Sigma Aldrich (Steinheim, Germany). GelStar™ Nucleic Acid Gel Stain was purchased from LONZA (Basel, Switzerland). Methanol was obtained from VWR (Radnor, Pennsylvania, PA, USA). eGFP expressing the DLD1 colon adenocarcinoma cell line was kindly provided by Dr. Thomas Müller and Dr. Jana Lützkendorf (Department for Internal Medicine IV (Oncology/Hematology), University Hospital Halle, Germany). DLD-1 cell line was purchased from American Type Culture Collection CCL-221^TM^ (ATCC, Manassas, VA, USA). Normal human dermal fibroblasts (NHDFs) and embryonic mouse fibroblasts (3T3) cells were kindly provided by Prof. Dr. Thomas Groth (Martin Luther University Halle-Wittenberg, Germany).

### 2.2. Methods

#### 2.2.1. Preparation of Liposomes

Liposomes were prepared following the method from J. Giselbrecht et al. 2019 with modifications in the final step [19]. Briefly, the ionizable lipid T14diLys and helper lipid DOPE stock solutions of 2 mg/mL in chloroform–methanol (8:2, *v*:*v*) were prepared separately and combined in a molar ratio of 1:2. For producing a thin film, the organic solvent was removed using a rotary evaporator (Büchi AG, Uster, Switzerland) for 30 min at 500 mbar and a further 1 h at ≤15 mbar. The film was dried overnight in the vacuum dryer (Binder GmbH & Co. KG, Hameln, Germany) to remove any solvent. On the next day, 1 mL of sterile 10 mM MES buffer pH 6.5 was added to the dry film to obtain a final concentration of 1 mg/mL. In the next step, the lipid dispersion was shaken at 1400 rpm at 50 °C for 30 min (Eppendorf thermomixer compact 5350). The modified final step was to extrude the dispersion through a 50 nm polycarbonate membrane 21 times to achieve unilamellar liposomes. The original last step, which was used to compare the methods, was to sonicate the dispersion at 37 kHz for 5 min at 30 °C.

#### 2.2.2. Preparation of Lipoplexes

Lipoplexes were produced as mentioned in J. Giselbrecht et al., 2019 [19]. The required amounts of siRNA and liposomes were combined and incubated for 15 min at 25 °C in 10 mM MES buffer pH 6.5. Thus, different N/P ratios are obtained depending on the type of experiment. The N/P ratio is the ratio of the positive charges of the primary amine group of the ionizable lipid to the negative charges of the phosphate groups of the siRNA.

#### 2.2.3. Size and Zeta Potential Measurement

The size and polydispersity index were determined by dynamic light scattering (DLS) using a Zetasizer Nano ZS (Malvern Instruments, Worcestershire, UK) with a scattering angle of 173°. Each sample was measured in triplicate at 25 °C using the intensity-weighted mode. Zeta potential measurements were performed by electrophoretic light scattering with the same device. Each sample was measured in triplicate in 10 mM MES buffer pH 6.5 at 50 V with 20 runs per measurement with a 30 s pause between 2 runs at 25 °C. The manufacturer’s software (Zetasizer software 7.12) was used for data analysis.

#### 2.2.4. Agarose Gel Electrophoresis

The required N/P ratio for total siRNA complexation was determined based on Nalbadis et al. 2021 with some modifications [10]. For this purpose, a 4% agarose gel containing 5 µL of GelStar^TM^ Nucleic Acid Gel Stain was prepared. Lipoplexes of different N/P ratios (0.6 µg siRNA in each sample) were prepared as mentioned earlier (Section 2.2.2). A volume of 10 µL glycerol/water solution 50% (*v*/*v*) was added to each lipoplex sample before loading them into the gel. GeneRuler Ultra Low Range DNA Ladder was added next to the samples into the gel. The electrophoresis was run for 1 h at 75 V in 1× TAE buffer pH 8. Afterward, the fluorescent bands were visualized and photographed using the CRi Maestro^TM^ fluorescence imaging system (CRi, Cambridge, MA, USA) and the Maestro software 2.10 with the blue filter set and automatic exposure times. The software ImageJ was used to analyze the fluorescence intensity of the bands.

#### 2.2.5. Transmission Electron Microscopy (TEM)

For negative staining, samples were diluted with 10 mM MES buffer (pH 6.5) to obtain N/P ratios 2–5 containing 0.6 µg siRNA per sample. For each sample, 3 µL were applied to a copper grid coated with a Formvar film. The excess liquid was drained off, and the samples were dried for 20 s. Next, samples were washed three times with water, and afterward, 2% aqueous uranyl acetate was added and removed after 1 min of incubation. The microscope EM900 (Carl Zeiss, Oberkochen, Germany) was used for sample examination at 80 kV. For the cryo-TEM images, 2 × 3 µL of each sample was applied on the holey carbon-grid in a sample chamber at 22 °C and 80% humidity. Afterwards, the samples were blotted for 10 s, and the grid was plunged in nitrogen-cooled liquid ethane. For sample observation, the Zeiss Libra 120 microscope (Carl Zeiss, Oberkochen, Germany) and 120 kV were used.

#### 2.2.6. Lipoplex Stability Test

For testing the lipoplex stability against RNase A, agarose gel electrophoresis was used. The gel production and running conditions were the same as described earlier in Section 2.2.4. After preparing lipoplexes at N/P 5 (0.6 µg siRNA per sample), they were incubated with 0.5 µL RNase A (c = 0.01 µg/µL) for 15 min at 37 °C. In the next step, RNase A activity was stopped by addition of 2.5 µL RiboLock RNase Inhibitor (c = 40 U/µL), followed by the same incubation conditions as before. An amount of 10 U/µL Heparin was added and slowly shaken at room temperature for 1 h (Eppendorf thermomixer compact 5350), and 10 µL glycerol/water solution 50% (*v*/*v*) was added before electrophoresis. Additionally, lipoplexes at N/P 5 were directly incubated with 10 U/µL Heparin at room temperature for 1 h and loaded into the gel. Lipoplexes at N/P 5 and non-complexed siRNA were also incubated with RNase A for 15 min at 37 °C. The fluorescence intensity of the bands was visualized and analyzed as shown in Section 2.2.4.

#### 2.2.7. Encapsulation Efficiency

The encapsulation efficiency of the lipoplexes was determined by RiboGreen-Assay using the Quant-iT^TM^ RiboGreen^TM^ RNA Kit (Invitrogen^TM^, Thermo Fisher Scientific Inc., Waltham, MA, USA) according to the manufacturer’s protocol. Lipoplexes of different N/P ratios and a positive control containing the same amount of siRNA (28,6 nM) as the lipoplex samples were prepared in triplicate in a black 96-well plate. Additionally, a calibration curve was added in duplicate. Afterward, 60 µL of 1× TAE buffer (pH 7.5) and 100 µL of 200-fold-diluted RiboGreen reagent were added to each well. Before the measurement, the plate was incubated in the absence of light for 5 min. The amount of unencapsulated siRNA was measured with the Cytation^TM^ 5 imaging reader (BioTek Instruments, Winooski, VT, USA) and the Gen5 3.12 software at an excitation wavelength of 485 nm and emission of 528 nm. The experiment was repeated three times independently.

The amount of encapsulated siRNA was calculated as shown below:

Encapsulation efficiency [%] = ((positive control siRNA − unencapsulated siRNA)/positive control siRNA) × 100(1)

#### 2.2.8. Cell Culture

DLD1 and the eGFP-DLD1 cell line were cultured in RPMI medium containing 10% FBS and 1% Pen/Strep. The 3T3 cells require DMEM with 10% FBS, 1% Pen/Strep, and 1 mM sodium pyruvate. NHDF cells need the same medium as 3T3 cells except for the sodium pyruvate. All cell lines were grown in an incubator at 37 °C with 5% CO_2_ and split 2 times per week.

#### 2.2.9. Transfection Experiments

In a 12-well plate, 1.3 × 10^5^ eGFP-marked DLD1 cells (eGFP-DLD1) per well were seeded and incubated for 24 h at 5% CO_2_ and 37 °C in RPMI medium supplemented with 10% FBS to obtain 30–50% confluency. The next day, lipoplexes at N/P 2–5 were prepared in 140 µL MES buffer 10 mM pH 6.5 by using an amount of 100 nM eGFP-siRNA per well. Meanwhile, the cell medium was changed to 500 µL serum-free RPMI. After 15 min, the lipoplexes were diluted in 360 µL OPTI-MEM^®^ Reduced Serum Medium and added to the cells. As a positive control, Lipofectamine2000^TM^ transfection reagent was combined with eGFP-siRNA and for the negative control with a scrambled siRNA. Additionally, the liposome concentrations without the siRNA were added as controls. The medium was changed to RPMI supplemented with 10% FBS 6 h later. After 72 h of total incubation time, the cells were photographed with the Cytation^TM^ 5 imaging reader (BioTek Instruments, Winooski, VT, USA) and the software Gen5 3.12. Afterward, the cells were trypsinated, harvested in tubes, and prepared for the eGFP quantification by flow cytometric analysis using the BD Accuri^TM^ C6 Plus Flow Cytometer (Becton, Dickinson and Company, Franklin Lake, NJ, USA). Immediately before the measurement, PI was added to obtain a final concentration of 2 µg/mL in each sample for viability control. A number of 10,000 events per measurement and sample were analyzed. All experiments were performed in duplicates on three different days with a new liposome batch each. The eGFP expression was calculated by setting the untreated cells to 100%.

For the filter test, either the liposomes were filtered through a 0.20 µm regenerative cellulose membrane, polyvinylidene fluoride membrane, or prepared under aseptic conditions before adding the siRNA.

#### 2.2.10. Cell Viability Assay

The resazurin reduction assay was performed to determine the cell viability at 24 h and 96 h after transfection. For this assay, the two fibroblast cell lines 3T3 and NHDF were used. For the NHDF, 20,000 cells per well for 24 h measurement and 2000 cells for 96 h were plated in a white 96-well plate with a clear bottom. For the 3T3 cells, 10,000 cells were seeded for 24 h and 3000 cells for 96 h per well. The first column was a blank, which only contained medium. The next day, 100 µL of Triton^TM^ X100 (0.05%) was added to the second column as a positive control. In the other columns, 100 µL of lipoplexes were added at three different RNA concentrations with N/P 5 and 10. Each column consists of 8 replicates. After 24 h and 96 h, 20 µL of resazurin (44 µM) was applied to each well and incubated for 2 h at 37 °C and 5% CO_2_ before fluorescence measurement. FI was measured using the Cytation^TM^ 5 imaging reader (BioTek Instruments, Winooski, VT, USA) and the software Gen5 3.12 (λ_ex_ = 531 nm, λ_em_ = 593 nm). Cell viability was determined by setting the untreated cells to 100% and subtracting the blank (without cells) from the results. Triton^TM^ X100 was added to achieve a cell viability of 0%. The assay was performed in quadruplicates on four different days.

#### 2.2.11. Statistical Analysis

Data are shown as mean ± standard deviation (SD). The software Origin 2019 was used for data analysis and IBM SPSS Statistics version 29.0.1.1 for creating the boxplot.

## 3. Results and Discussion

### 3.1. Characterization of Liposomes and Lipoplexes

**Figure 1:**
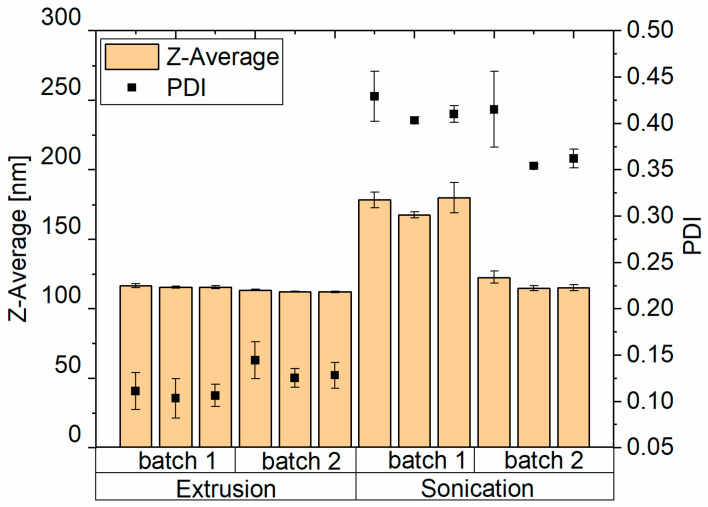
Impact of the manufacturing process on Z-Average and Polydispersity index (PDI) between T14diLys:DOPE (1:2) liposome (0.05 µg/µL) preparation with extrusion and sonication in 10 mM MES buffer pH 6.5, three samples per batch with three measurements each.

The extrusion method is a generally known method for achieving small, homogenous unilamellar liposomes. Additionally, the process has a high reproducibility. We therefore chose this technique as a potential way of improving the particle properties compared to the originally used sonication. The particle properties of sonicated and extruded liposomes were investigated regarding differences in size, polydispersity, and reproducibility. As seen in Figure 1, the liposome size and Polydispersity Index (PDI) after sonication are bigger and show higher variability compared to the extruded ones. The sonicated liposomes have a PDI of 0.396 ± 0.03, which indicates a wide size distribution of the particles. Additionally, the particle size is different when comparing different batches and, at an average of 146 ± 4 nm, is higher than the particles obtained with the extrusion method. The extruded particles have a size of 114.1 ± 1.9 nm, and the PDI is 0.119 ± 0.02 (Figure 1). Due to the improved batch-to-batch reproducibility of the liposome size and the smaller PDI when using extrusion, this method was used for all further studies.

**Figure 2:**
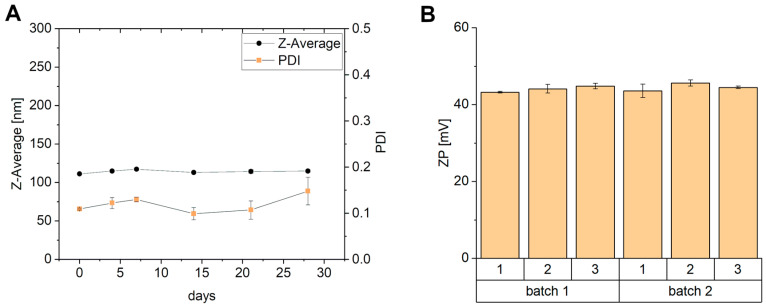
(**A**) Impact of storage time on size and polydispersity for 28 days of T14diLys:DOPE (1:2) liposomes (0.05 µg/µL) prepared with extrusion in 10 mM MES buffer pH 6.5, n = 3. The error bars are within the limits of the symbols. (**B**) Results of zeta potential of three T14diLys:DOPE (1:2) liposome (0.6 µg/µL) samples of two batches in 10 mM MES buffer pH 6.5, n = 3.

To obtain information about the stability of the liposomes during a longer period, the liposomes were stored at 4 °C for 28 days, and the size and PDI were measured on days 0, 4, 7, 14, 21, and 28 (Figure 2A). No major changes in size and PDI can be observed. In terms of liposome stability, the zeta potential needs to be considered as well. The zeta potential of the liposomes is above 30 mV and does not change from batch to batch (Figure 2B). The high stability of the dispersion can be explained by the repulsive forces of the particles [28,29].

**Figure 3:**
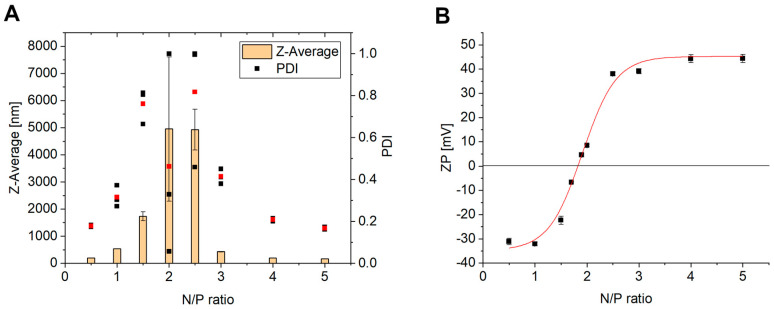
Impact of N/P ratio on particle size, polydispersity (**A**), and zeta potential (**B**) in 10 mM MES buffer pH 6.5 of T14diLys:DOPE (1:2) lipoplexes (5 ng/µL siRNA per sample), n = 3. Red dots correspond to the mean value of the three black dots, which represents the PDI.

**Figure 4:**
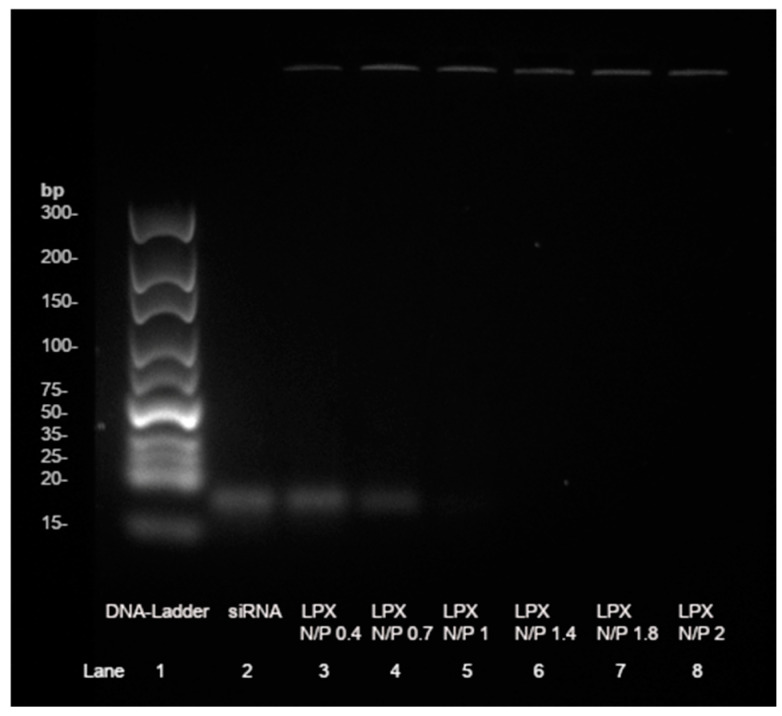
Separation of lipoplex preparations in agarose gel electrophoresis as a function of different N/P ratios: The complexation efficiency of siRNA by T14diLys:DOPE (1:2). Lipoplex = LPX. At NP ratios > 1, no band visible.

**Figure 5:**
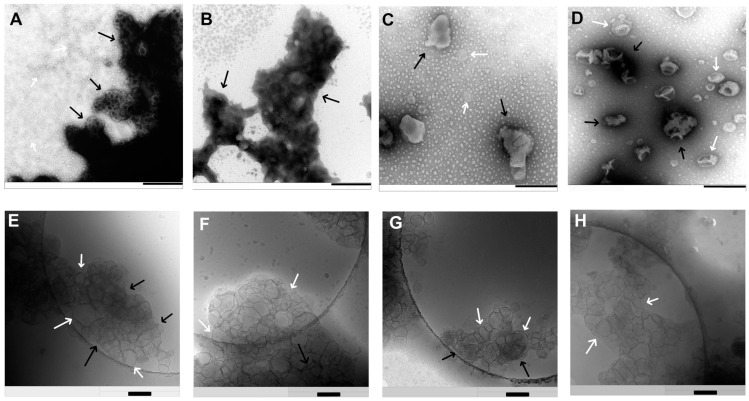
TEM images of T14diLys:DOPE (1:2) lipoplexes in 10 mM MES buffer pH 6.5 after adding uranyl acetate for negative staining at N/P 2 (**A**), N/P 3 (**B**)**,** N/P 4 (**C**), and N/P 5 (**D**)—scale bar in (**A**,**B**) represents 500 nm—and (**C**,**D**) 250 nm Cryo-TEM images of T14diLys:DOPE (1:2) lipoplexes in 10 mM MES buffer pH 6.5 at N/P 2 (**E**), N/P 3 (**F**), N/P 4 (**G**), and N/P 5 (**H**)—scale bar in (**E**–**H**) represents 200 nm. Black arrows indicate for lipoplexes, white arrows for uncomplexed liposomes.

The extruded liposomes can be combined with siRNA in different N/P ratios to obtain lipoplexes. For this purpose, the siRNA concentration has kept constant, and the amount of the lipid varied. For effective protection and transfection, the siRNA needs to be at least fully complexed by the positively charged liposomes. Therefore, the point of complete complexation has to be determined. For this purpose, zeta potential measurements and agarose gel electrophoresis were used. When measuring the zeta potential of lipoplexes with increasing N/P ratios, the Isoelectric Point (IEP) can be determined (Figure 3B). At that point of 0 mV, the RNA is completely complexed and there are no longer enough repulsive forces to prevent particle agglomeration. This can be seen at N/P 1.83, where the red-fitted sigmoidal curve crosses the 0 mV with a strong gradient of the curve. The reason for this is that all negative charges of the phosphate backbone of the siRNA are complexed with the ionizable liposomes, which is consistent with the result from agarose gel electrophoresis (Figure 4). When the nucleic acid stain intercalates in the siRNA, a band can be detected by fluorescence. In the gel, the siRNA bands become weaker and weaker with an increasing amount of liposomes until no band can be seen and detected at N/P 1.8 (Figure 4, lane 7) [30]. At this point of full complexation, the negatively charged siRNA can no longer pass through the gel to the positive end. Therefore, the siRNA complex remains in the start line. In comparison, a band with naked siRNA as a control is visible in lane 2 [31]. The IEP at NP 1.83 also explains the large increase in size and PDI at N/P 2 and 2.5 (N/P 2 = 4950.0 nm; N/P 2.5 = 4926.7 nm) (Figure 3A). If the amount of liposomes is increased further to N/P 3, 4, and 5, it can be seen that the particle size and distribution become much smaller again. At N/P 3, the particle size decreases to 426.4 nm, but the PDI is still high at 0.414. Lipoplexes at N/P 5 resulted in small particles (Z-average = 162.3 nm) with a narrow size distribution (PDI = 0.168) (Figure 3A) and were thus promising for successful gene knockdown. Since a local delivery system is planned with this formulation, a positive charge of the lipoplexes is beneficial due to adsorption and desorption effects [32]. The details of the lipoplex structure at N/P ratios 2–5 were obtained by electron microscopy using negative staining-TEM (Figure 5A–D) and cryo-TEM technologies (Figure 5E–H). Both liposomes and lipoplexes can be seen in the negative staining micrographs. The lipoplexes appear dark black due to the interaction of negatively charged siRNA with uranyl-ions (Figure 5C,D (black arrows)) [18,19]. The uncomplexed liposomes, on the other hand, are light in color (white arrows) [18,19]. The differences between the various N/P ratios are interesting. At N/P 2 and 3, the lipoplexes are attached to each other and form large aggregates (black arrows), which results in a dark contrast due to the higher mass density. This aggregation is consistent with the zeta potential and DLS measurements (Figure 5A,B compared to Figure 3A,B). It is also possible that siRNA is increasingly localized on the surface in the transition area and has an electrostatic aggregation effect on the positive lipid charges on the surface. It is therefore possible that, despite a positive zeta potential, there are both positively and negatively charged domains on the surface. At N/P 4 and 5, the dark lipoplexes can be seen separately, and the liposomes are clearly recognizable next to them (Figure 5C,D). The characteristic lamellar structures of the lipoplexes are well visible here as well. When looking at the cryo-TEM images, there are a lot of attached liposomes with different sizes observable, probably due to the sample preparation (white arrows). The lamellar structures between two liposomes and some concentric lamellar ones represent lipoplexes (black arrows), which can be found in Figure 5E–G [33,34]. These structures possibly arise from an alternating layering of siRNA and liposomes [33]. It is not completely clear if the smaller liposomes are inside bigger ones or behind each other.

When preparing lipoplexes, a high encapsulation of the siRNA is always aimed for. Thus, the encapsulation efficiency of the lipoplexes was also investigated using the RiboGreen-Assay according to the manufacturer’s protocol. In brief, RiboGreen reagent is an ultrasensitive fluorescent nucleic acid dye that shows a fluorescent signal when bound to free and intact RNA, including siRNA [35]. Specifically, for lipid nanoparticles (LNPs), 0.5% Triton^TM^ X100 is added to the LNPs to release all of the RNA of the particle so that the total amount of RNA can be determined [30,36,37]. However, when using 0.5% Triton^TM^ X100 with an incubation time of 15 min, it was not possible to destroy the lipoplexes and release all the siRNA completely. This problem also occurs when the incubation time was extended to 2 h or the Triton concentration was raised to 2%. Therefore, the non-encapsulated siRNA and siRNA on the outside of the liposomes were determined with RiboGreen and compared with a positive control containing the same amount of siRNA as in the lipoplexes. Even though N/P 2 and 3 do not have adequate size and stability properties, it is unclear if this affects the encapsulation efficiency. The highest encapsulation was found at N/P 5 with 98.8% ± 0.6 (Table 1). N/P 2–4 show high encapsulation efficiencies, although they have poorer particle properties. Thus, just a tiny amount of siRNA is outside or not bound to the lipoplex and not protected from degradation by enzymes.

### 3.2. Lipoplex Stability Test

**Figure 6:**
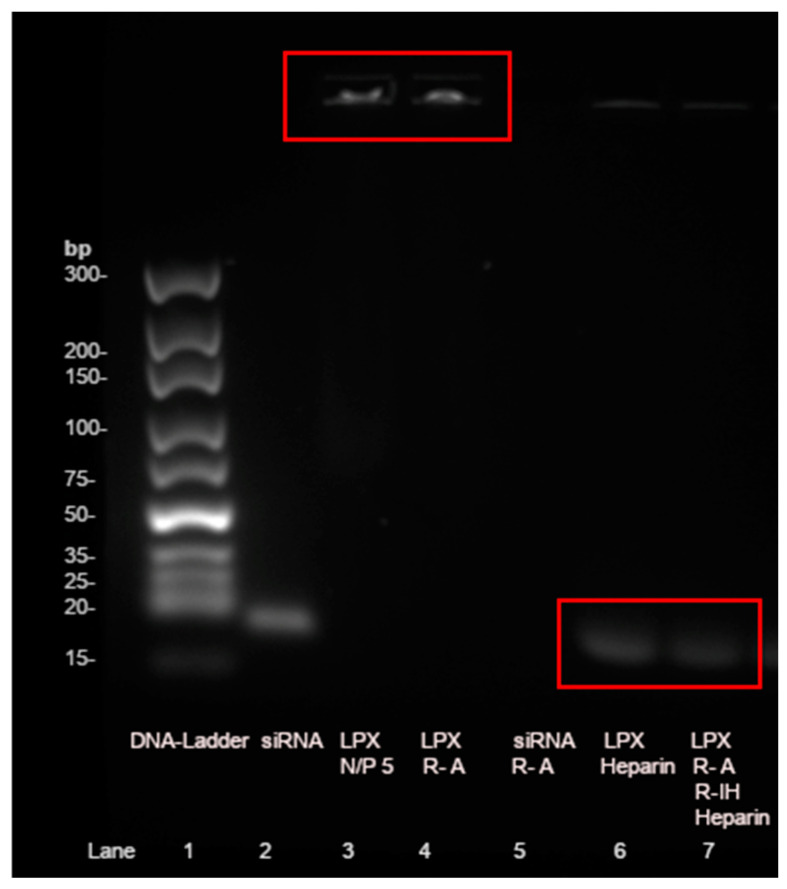
Agarose gel electrophoresis chromatogram of T14diLys:DOPE (1:2) lipoplexes (LPX) at N/P 5 after incubation with RNase A (R-A) and release of stable siRNA out of lipoplex with Heparin after RNase A and RNase Inhibitor (R-IH) treatment. The red boxes highlight the important bands.

One of the most important challenges of siRNA delivery is the degradation by endogenous nucleases, e.g., RNase A in the body [38]. In this assay, the ability of the lipoplexes to protect siRNA against RNase A was tested (Figure 6). Therefore, the lipoplexes at N/P 5, the most interesting ratio, and non-complexed siRNA were incubated with RNase A and characterized by agarose gel electrophoresis. Non-complexed negatively charged siRNA runs through the gel to the positive end of the chamber (lane 2). When adding the liposomes to the siRNA at an N/P ratio of 5, the siRNA cannot run through the gel and stays in the start line (lane 3). After incubation of the lipoplexes with RNase A for 15 min at 37 °C, the intensity of the start line (lane 4) is still the same compared to lane 3. That indicates that the lipoplex can protect the RNA from degradation, especially when comparing those with lane 5. Lane 5 shows the addition of RNase A to naked siRNA. No band is visible due to RNA degradation [39]. To release the siRNA from the lipoplex, it can be incubated with heparin for 1 h at room temperature. Heparin is a polyanion that competes with siRNA in the lipoplex and thus releases RNA from the complex [40]. Therefore, a band is visible in lane 6. This lane is slightly weaker than the naked siRNA control (lane 2), probably due to some interactions of the heparin with the lipid components [41]. In lane 7, lipoplexes were first incubated with RNase A, then an RNase inhibitor (RiboLock RNase Inhibitor) was added (to stop the RNase activity) and incubated for 15 min at 37 °C, and finally heparin released the siRNA. Despite RNase treatment, RNA can be released from the complex (lane 7). However, there is a 25% decrease in band intensity in lane 7 after the RNase treatment compared to lane 6. This may be caused by enzymes interfering with the heparin decomplexation or by 25% of the siRNA being accessible to RNase degradation. The results of lanes 4 and 7 show that the lipoplexes can partially protect the siRNA in the presence of RNase A.

### 3.3. GFP-Knockdown Efficiency

**Figure 7:**
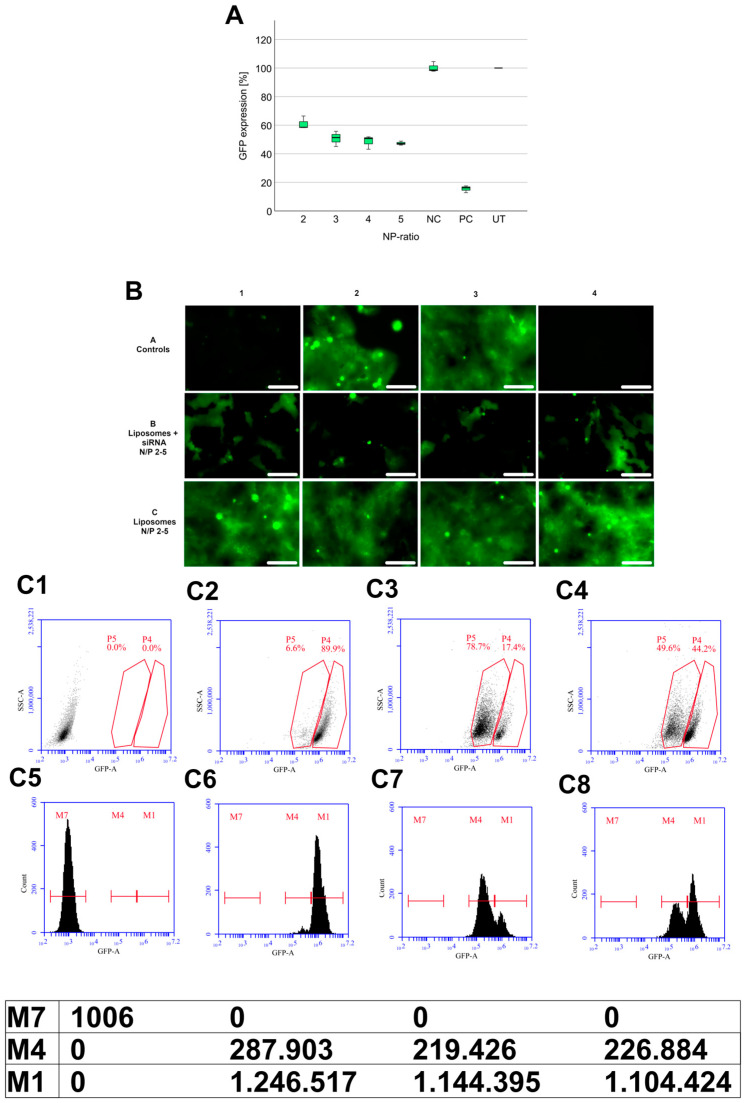
(**A**) Boxplot of eGFP expression level of (N/P 2–5) T14diLys:DOPE (1:2) lipoplexes at different N/P ratios after 72 h incubation (100 nM siRNA); (NC) negative control = scrambled siRNA + Lipofectamine2000 and (PC) positive control = eGFP siRNA with Lipofectamine2000 and (UT) untreated eGFP-DLD1 cells. eGFP expression was analyzed as duplicates. The experiment was repeated 3 times independently. Untreated eGFP-DLD1 cells are set to 100%. (**B**) Fluorescence images with Cytation 5 after 72 h from transfection of eGFP-DLD1 cells, A1 eGFP siRNA with Lipofectamine2000, and scrambled siRNA with Lipofectamine2000 (A2) with Lipofectamine2000, A3 untreated eGFP-DLD1 cells, A4 wildtype DLD1 cells. B1–4 T14 diLys:DOPE (1:2) lipoplexes N/P 2–5, C1–4 liposome amount N/P 2–5. 200 × zoom, scale bars = 100 µm. (**C**): Flow cytometry data as dot plots (**C1**–**C4**) and histograms (**C5**–**C8**) from (**C1** + **C5**): wildtype DLD1 cells, (**C2** + **C6**): eGFP-DLD1 cells, (**C3** + **C7**): eGFP-DLD1 cells with Lipofectamine2000 + siRNA, (**C4** + **C6**): eGFP-DLD1 cells with T14diLys:DOPE (1:2) + siRNA N/P 5. M7, 4, and 1 represent the GFP intensity in (**C5**–**C8**).

After characterizing the lipoplexes, the ability to knock down the eGFP was tested via eGFP-DLD1 cells. Lipoplexes with an N/P ratio of 2–5 were also selected here. These ratios were used to see whether size, zeta potential, and N/P ratio influence the transfection efficiency. Lipoplexes at N/P ratio from 2 to 5 containing T14diLys:DOPE (1:2) liposomes were able to reduce the eGFP expression to 61.0% ± 4.6 (N/P 2) − 47.1% ± 4.3 (N/P 5) compared to untreated cells (Figure 7A). There is a slight decrease in eGFP expression from N/P 2 to 5. The N/P ratio of 5 has the largest impact on the reduction of the eGFP expression. The dot plots obtained by flow cytometric analysis (Figure 7(C1–C4)) show the DLD1 cells with low FI (Fluorescence intensity) (Figure 7(C1)) and the eGFP-DLD1 cells with a signal that is shifted to higher FI values (Figure 7(C2)). When applying eGFP-siRNA-containing lipoplexes to the eGFP-DLD1 cells, the signal shifts again down to lower FI (Figure 7(C3,C4)). These results can also be seen in the images taken by the Cytation^TM^ 5 imaging reader in Figure 7B. The image with the untreated eGFP-DLD1 cells (A3) is completely green after 72 h. Additionally, the images in the last line C, where just the liposomes without siRNA were added, are also intensive green. This indicates that the liposomes themselves have little or no toxic effects on the eGFP-DLD1 cells, otherwise, the cells would not grow so strongly. The liposomes without siRNA have no knockdown effect. Figure 7B, image A1, is the positive control containing Lipofectamine2000 as a gold standard transfection reagent [42]. The eGFP expression goes down to 15.4% ± 1.7, which explains the almost black image. In line B1–4, the lipoplexes containing T14diLys/DOPE (1:2) liposomes at N/P 2–5 were added. It can be seen that just some parts are green, but most of it is black. The black parts are the cells where the eGFP is downregulated, and in the green parts, the protein is still expressed and shows a fluorescent signal. In conclusion, the N/P ratio of 5 has the highest transfection efficiency.

**Figure 8:**
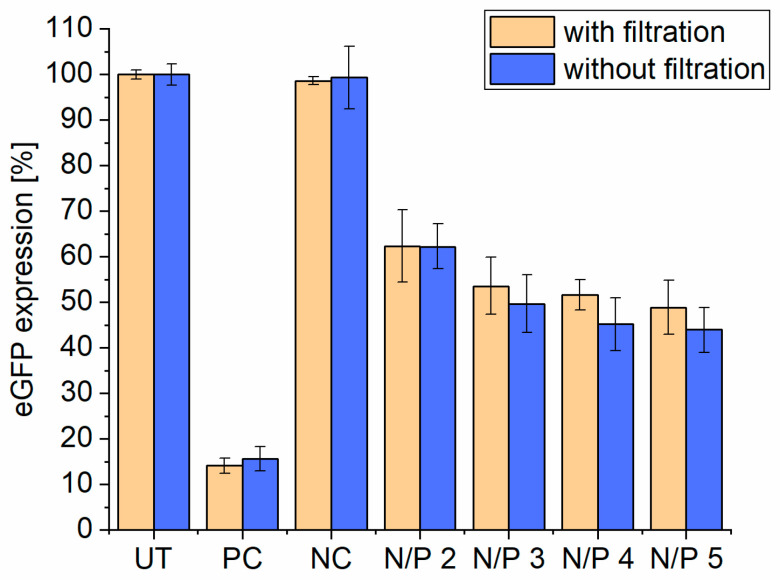
eGFP expression level of eGFP-DLD1 cells at different N/P ratios (100 nM siRNA) after 72 h incubation with and without sterile filtration of T14diLys:DOPE (1:2) liposomes before transfection using 0.20 µm regenerative cellulose filter membrane. The (NC) negative control = scrambled siRNA + Lipofectamine2000 and (PC) positive control = eGFP-siRNA were combined with Lipofectamine2000, (UT) untreated eGFP-DLD1 cells. eGFP expression was analyzed as duplicates. The experiment was repeated 3 times independently. Untreated eGFP-DLD1 cells are set to 100%.

We also investigated if it is possible to perform a sterile filtration of the liposomes before the addition of siRNA. Different filter materials with 0.20 µm pore size were tried out: PVDF and RC. When filtrating the liposomes with PVDF filters, DLS measurements showed no particles anymore. The particles are probably retained by the filter material through unspecific binding. These problems did not occur with RC filters, because after filtration, particles with the same attenuator could be detected with DLS. Therefore, the RC filtrated liposomes were tested in combination with siRNA and lipoplexes at N/P 2–5 on the eGFP-DLD1 cells to see if there is a difference in knockdown efficiency to non-filtrated ones. As shown in Figure 8, there is no major difference in knockdown efficiency. This leads to the conclusion that only a neglectable amount of liposomes are retained in the RC filter material. We also tested the transfection efficiency with PVDF-filtrated liposomes, but no eGFP knockdown could be obtained. Therefore, the liposomes can be sterile-filtered without any problems before transfection with a 0.20 µm RC filter.

**Figure 9:**
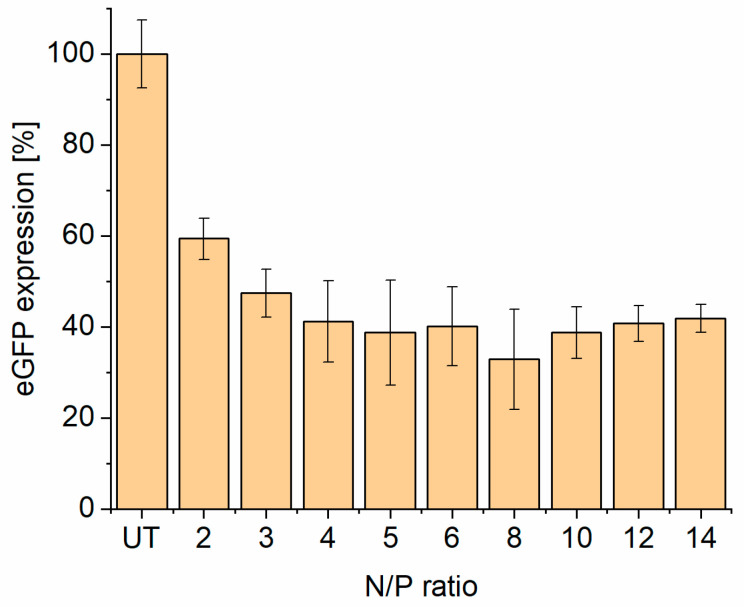
eGFP expression level at increasing N/P ratios (100 nM siRNA) of T14diLys:DOPE (1:2) lipoplexes after 72 h incubation; UT = untreated eGFP-DLD1 cells.

In the previous in vitro experiments, there was a slight increase in the transfection efficiency visible toward N/P 5. Therefore N/P ratios higher than 5 where selected as well to investigate if the knockdown efficiency would increase further. For this attempt, N/P ratios of 2 to 14 were applied to the eGFP-DLD-1 cells, and again the eGFP expression was measured after 72 h using flow cytometry. Figure 9 shows a plateau starting at N/P 5. At higher amounts of liposomes, the knockdown efficiency does not increase remarkably, but it is possible that the toxicity could rise, as this is the case for cationic liposomes [9,43]. The N/P ratio with the highest efficiency and the lowest amount of liposomes possible, N/P 5, was chosen for further experiments. These results match the previous results from zeta potential measurement. At N/P ratios greater than 3, we also had a zeta potential plateau (Figure 3B). Even if the number of liposomes increases, the charge of the lipoplex did not increase extremely and therefore not the transfection efficiency.

### 3.4. Cytotoxicity

**Figure 10:**
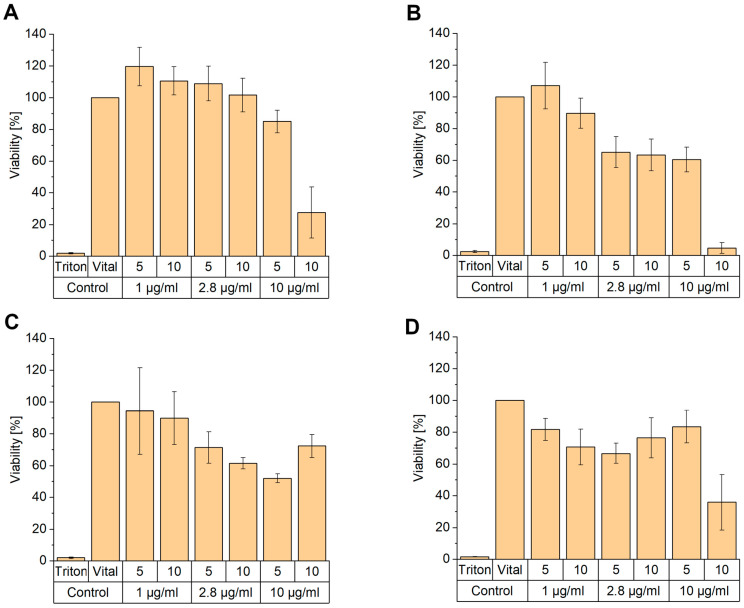
Results of cytotoxicity of T14diLys/DOPE (1:2) lipoplexes at N/P 5 and 10 with siRNA concentrations of 1, 2.8, and 10 µg/mL after 24 h and 96 h incubation in NHDF and 3T3 cells. (**A**) NHDF after 24 h, (**B**) NHDF after 96 h, (**C**) 3T3 after 24 h, (**D**) 3T3 after 96 h. The vital control contains just cells and medium representing the 100% vital cells, and the positive control includes also Triton X100, which represents the most toxic effect possible. Results are presented as mean ± SD, n = 4.

Ionizable liposomes are an effective option for siRNA delivery, but the in vitro and in vivo toxicity, especially for permanently cationic ones, has to be considered [44,45]. N/P 5 is the most interesting ratio due to the particle properties, highest knockdown efficiency, and encapsulation efficiency. Therefore, the cytotoxicity of this ratio and N/P 10 was analyzed using the resazurin assay in the sensitive 3T3 and NHDF cells at three different siRNA concentrations (1, 2.8, and 10 µg/mL) after 24 h (acute toxicity) and 96 h (long-term toxicity). N/P 10 was also chosen to see if higher ratios and higher amounts of ionizable lipids would cause toxicity. After 24 h of lipoplex incubation, there is no decrease in cell viability in NHDF at siRNA concentrations of 1 and 2.8 µg/mL at both N/P ratios visible (Figure 10A). At concentrations of 10 µg/mL, the cell viability at N/P 5 (85.0% ± 7.2) is slightly lower, but at N/P 10, acute toxicity is visible. For the long-term toxicity test, just the samples of 1 µg/mL at both N/P ratios have no toxic effects compared to the vital control (Figure 10B). However, especially 10 µg/mL and N/P 10 decrease the NHDF viability to 4.7% ± 3.4. The viability of NHDF after adding lipoplexes at N/P 10 is always at least slightly lower than at N/P 5. Although toxicity at higher concentrations is mainly known for cationic liposomes, this is also visible here in the case of an ionizable lipid [43]. The results from 3T3 cells differ from the NHDF results. After 24 h, 3T3 cells are at 1 µg/mL, and both N/P ratios demonstrate a small decrease in cell viability (Figure 10C) compared to Figure 10A. The higher concentrations reduce the vital 3T3 cells up to 52.0% ± 2.8 at 10 µg/mL at N/P 5 compared to the NHFD at 24 h (85.0% ± 7.2). The reason might be that 3T3 cells are probably more sensitive compared to NHDF [46,47]. Surprisingly, the highest concentration at N/P 10 has higher cell viability values than the two samples with lower lipid amounts (Figure 10C). This could be due to a boost in the cell metabolism [46]. After 96 h, the cell viability creates a U-shape with a minimum of 2.8 µg/mL N/P 5 and 66.7% ± 6.2 viability, except from the highest RNA and liposome concentration (Figure 10D). It may be possible that the 3T3 cells recover from the lipoplexes over a longer period, except for the highest concentration, which has clear toxicity. In conclusion, the combination of siRNA concentration 1 µg/mL and N/P 5 has the smallest impact on cytotoxicity in 3T3 and NHDF cell lines.

## 4. Conclusions

The results of our study indicate that the lipid combination T14diLys: DOPE (1:2, n/n) proved to be effective for siRNA delivery. Second, the extrusion process improved the quality of the liposomes by producing smaller particles with a narrower size distribution. Lastly, the obtained liposomes and lipoplexes were tested in terms of stability, encapsulation, and knockdown efficiency and toxicity. Our study showed that for lipoplexes of this lipid mixture, the N/P ratio 5 is the most promising ratio for a possible local siRNA delivery system in the future.

## Figures and Tables

**Table 1:** Encapsulation efficiency of T14diLys:DOPE (1:2) lipoplexes using RiboGreen-Assay, analyzed in triplicates with 3 independent experiments.

N/P Ratio	Encapsulation Efficiency [%] ± SD
2	94.1 ± 3,7
3	97.6 ± 1.3
4	98.4 ± 0.7
5	98.8 ± 0.6

## Data Availability

The data will be made available on request.
